# Xanthohumol Inhibits the Growth of Keratin 18-Overexpressed Esophageal Squamous Cell Carcinoma *in vitro* and *in vivo*

**DOI:** 10.3389/fcell.2020.00366

**Published:** 2020-05-19

**Authors:** Shuying Yin, Mengqiu Song, Ran Zhao, Xuejiao Liu, Woo Kyu Kang, Jeong Min Lee, Young Eun Kim, Chengjuan Zhang, Jung-Hyun Shim, Kangdong Liu, Zigang Dong, Mee-Hyun Lee

**Affiliations:** ^1^Department of Pathophysiology, School of Basic Medical Sciences, Zhengzhou University, Zhengzhou, China; ^2^China-US (Henan) Hormel Cancer Institute, Zhengzhou, China; ^3^Department of Neurobiology, University of Massachusetts Medical School, Worcester, MA, United States; ^4^Department of Biochemistry and Molecular Pharmacology, University of Massachusetts Medical School, Worcester, MA, United States; ^5^Therapeutics & Biotechnology Division, Korea Research Institute of Chemical Technology, Daejeon, South Korea; ^6^The Affiliated Cancer Hospital, Zhengzhou University, Zhengzhou, China; ^7^Department of Pharmacy, College of Pharmacy, Mokpo National University, Mokpo-si, South Korea; ^8^College of Korean Medicine, Dongshin University, Naju, South Korea

**Keywords:** xanthohumol, keratin18, esophageal squamous cell carcinoma (ESCC), protein stability, cell growth

## Abstract

Esophageal squamous cell carcinoma (ESCC) is a leading cause of cancer-related death worldwide. Xanthohumol is a prenylated flavonoid isolated from *hops*. Although xanthohumol has been reported to exert anti-obesity, hypoglycemic, anti-hyperlipidemia and anti-cancer activities, the mechanisms underlying its chemotherapeutic activity are yet to be elucidated. In the present study, we found that xanthohumol inhibited ESCC cell proliferation *in vitro* and *in vivo* by targeting keratin (KRT)-18. Xanthohumol suppressed the proliferation, foci formation, and anchorage-independent colony growth of KYSE30 cells. Using xanthohumol-sepharose conjugated bead pull-down and mass/mass analysis, we found that KRT18 is a novel target of xanthohumol in KYSE30 cells. KRT18 protein was highly expressed in patient ESCC tissues compared to adjunct tissues. Anti-proliferative activity of xanthohumol was abrogated or enhanced according to the knockdown or overexpression of KRT18 protein, respectively. Xanthohumol also induced apoptosis and cell cycle arrest at G1 phase which was associated with the modulation of expression of related makers including cyclin D1, cyclin D3, and cleaved-PARP, Bcl-2, cytochrome c and Bax. While xanthohumol attenuated KRT18 protein expression, it failed to cause any change in the KRT18 mRNA level. Furthermore, oral administration of xanthohumol decreased tumor volume and weight in patient-derived xenografts (PDXs) tumors having overexpressed KRT18. Overall these results suggest that xanthohumol acts as a KRT18 regulator to suppress the growth of ESCC.

## Introduction

Esophageal cancer (EC) is the ninth leading cause of cancer-related death worldwide. EC patients always have poor prognosis with 5-year survival rate about 19% (Bray et al., 2018; Siegel et al., 2019). Off the two primary subtypes of EC, the esophageal squamous cell carcinoma (ESCC) has 70% prevalence globally with predominant distribution in the developing countries located in eastern Asia and parts of Africa (Smyth et al., 2017), and the esophageal adenocarcinoma (EAC) is mostly prevalent in Western countries (Testa et al., 2017). Cancer Statistics in China reports that EC ranks the third in men and fifth in women among all diagnosed cancer (Chen et al., 2016). Major risk factors of ESCC includes but are not limited to tobacco smoking, chronic alcohol consumption, and exposure to dietary carcinogens (Yang et al., 2016; Lagergren et al., 2017).

About 19% of patients with EC are diagnosed at early localized stage when endoscopic resection or endoscopic ablation can cure the disease (Ahmed et al., 2019). Combination of surgery with chemotherapy and/or radiotherapy is the common method for treating EC and improve the survival of patients (Mayanagi et al., 2019). The commonly used chemotherapy in clinical settings include cisplatin, 5-fluorouracil, docetaxel, carboplatin, paclitaxel, and bleomycin as well as their combinations (Nakamura et al., 2013; Ku, 2017). Targeted therapies and immunotherapies are also two emerging therapy used to treat ECs. Although Food and Drug Administration (FDA)-approved 13 drugs for EC treatment, many of these are targeted therapies such as cetuximab and lapatinib (targeting epidermal growth factor receptor, EGFR), irinotecan (inhibitor of type I topoisomerase), trastuzumab (monoclonal antibody against human epidermal growth factor receptor 2, HER2) and apitinib (inhibitor of vascular endothelial growth factor, VEGFR) (Teicher, 2008; Davidson and Starling, 2016; Huang et al., 2018; Barsouk et al., 2019). However, these drugs have failed to reach clinical success to curb EC prevalence largely due to non-responsiveness of many patients. There are also many investigational drugs targeting MET, mammalian target of rapamycin (mTOR), or poly ADP ribose polymerase (PARP), which have been failed in phase II or phase III clinical trial (Lyons and Ku, 2017; Liu X. et al., 2019). The immunotherapy agent pembrolizumab, has been approved by the FDA to treat PD-L1-positive metastatic EAC and ESCC in July 2019, but patients contained PD-L1 expression is not so high to response to this agent (Barsouk et al., 2019). Thus, there is emerging need for developing new therapies with better efficacy and tolerability by focusing on novel drug targets.

Keratin 18 (KRT18) is a member of the intermediate filament family of cytoskeletal protein, it plays an important role for tissue integrity (Lebherz-Eichinger et al., 2013). KRT18 was found to be over expressed in many different types of cancer including that of colorectal cancer and associated with tumor stage, cancer migration, and invasion (Kilic-Baygutalp et al., 2016). The relatively higher serum KRT18 levels in ESCC patients as compared to normal subjects were correlated with tumor progression (Kilic-Baygutalp et al., 2016).

Because of multiple biological activities and relatively low systemic toxicity, natural compounds gained much attention from researchers to explore its anticancer function (Song et al., 2018; Liu X. et al., 2019). Xanthohumol, a prenylated flavonoid isolated from hops (*Humulus lupulus* L.), has anti-obesity, hypoglycemic and anti-hyperlipidemia activities (Liu et al., 2015; Jiang et al., 2018). Numerous *in vitro* and *in vivo* studies have revealed the anticancer effect of xanthohumol (Sun et al., 2018; Wei et al., 2018; Liu W. et al., 2019; Slawinska-Brych et al., 2019). Xanthohumol exerts anti-cancer effects through inhibition of the activity of AKT, mTOR, NFκB/IKK, IL-1β and TNFα (Guo et al., 2018; Li et al., 2018; Saito et al., 2018; Lin et al., 2019; Liu X. et al., 2019). Previously we have reported that xanthohumol inhibited the growth of AKT kinase overexpressing ESCC cells (Liu X. et al., 2019). We also found that xanthohumol could inhibit the proliferation of cells with low level of AKT. This led us to continue to find additional molecular target of xamthohumol for its anti-cancer effects. Mass spectrometry analysis Revealed that xanthohumol binds to KRT18 protein. We, therefore, examined whether xanthohumol can elicit anti-cancer effects via modulation of KRT8. Here we report that xanthohumol inhibits ESCC cell proliferation and colony formation through the induction of cell cycle arrest at G1 phase and apoptosis, which was associated with decreased expression of KRT18. Moreover, xanthohumol inhibits the growth of KRT18 overexpressing ESCC patient-derived xenograft (PDX) tumors in mouse model.

## Materials and Methods

### Reagents

Xanthohumol (purity ≥ 97%) was purchased from Sichuan Weikeqi Biological Technology, Co., Ltd. (Chengdu, China). Antibodies to detect Keratin18 (ab668) was purchased from Abcam (Cambridge, MA, United States). Antibodies to examine Bcl-2 (CST 15071), Bax (CST 5023), cyclin D1 (CST 2922), cyclin D3 (CST 2936), COX IV (CST 4850), α-tubulin (CST 3873), and β-tubulin (CST 5346) expression was from Cell Signaling Technology (Beverly, MA, United States). Antibodies to detect β-Actin (sc-47778) and cytochrome c (sc-13156) were from Santa Cruz (Santa Cruz, CA, United States). Goat anti-rabbit antibody (ZB-2301) and goat anti-mouse antibody (ZB-2305) were obtained from ZSGB-Bio Company (Beijing, China).

### Cell Culture

The human EC cell line KYSE30, KYSE70, KYSE410, KYSE450, and KYSE510 was purchased from the Type Culture Collection of the Chinese Academy of Sciences (Shanghai, China). Cells were cultured in RPMI-1640 containing penicillin (100 units/mL), streptomycin (100 μg/mL), and 10% fetal bovine serum (FBS, Biological Industries, Kibbutz Beit-Haemek, Israel). The human immortalized normal esophageal epithelial cell line, SHEE, was donated by Dr. Enmin Li from the Laboratory of Tumor Pathology (Shantou University Medical College, Shantou, China) (Shen et al., 2002). Cells were maintained in a humidified atmosphere at 37°C, contain 5% CO_2_. Cells were cytogenetically tested and authenticated before being frozen. Each vial of frozen cells was thawed and maintained in culture for a maximum of eight passages.

### Cell Proliferation Assay

Cells (1.2 × 10^3^ cells/well) were seeded in 96-well plates and incubated for 24 h, then treated with different doses of xanthohumol or DMSO (dimethyl sulfoxide, Sigma-Aldrich, St. Louis, MO, United States). Then measured cell proliferation using MTT [(4,5-dimethylthiazol-2-yl)-2,5-diphenyltetrazolium bromide, Ruitaibio, Beijing, China] agents at 24, 48 or 72 h. For foci formation assay, cells (1.2 × 10^3^ cells/well) were seeded in 6-well plates and incubated for 24 h, then treated with different doses of xanthohumol or vehicle. After a week culture, stained with crystal violet (Solarbio, Beijing, China) and count the foci number. For anchorage-independent cell growth, cells (8 × 10^3^ cells/well) suspended in complete medium and 0.3% agar with different concentration of xanthohumol or vehicle, with a base layer of 0.5% agar contain different concentration of xanthohumol or vehicle. Then cultured at 37°C in a 5% CO_2_ incubator for 2 weeks and visualized the colony under a microscope and counted using the Image-Pro Plus software (v.6.1) program (Media Cybernetics, Rockville, MD, United States).

### Cell Cycle Analysis

KYSE30 cells (2 × 10^5^) were seeded into 60-mm plates and 24 h later, treated with xanthohumol or vehicle for 24 h. Cells were fixed in 70% ethanol and stored at −20°C for 24 h. After staining with propidium iodide at room temperature (RT) for 15 min, analyzed cell cycle distribution using a BD FACS Calibur Flow Cytometer (BD Biosciences, San Jose, CA, United States).

### Annexin V Apoptosis Assay

Cells (2 × 10^5^) were seeded in 60-mm plates and incubated for 24 h then treated with xanthohumol or vehicle for 48 h. Cells were stained with annexin-V and propidium iodide at RT for 15 min. Apoptotic cells were analyzed using a BD FACS Calibur Flow Cytometer (BD Biosciences, San Jose, CA, United States).

### Western Blot Assay

Cell pellets were lyzed in cell lysis buffer containing 50 mM Tris pH 8.0, 0.5–1% NP-40, 150 mM NaCl, protease inhibitor cocktail, and 1 mM PMSF. Supernatant fractions were collected after centrifuge at 14,000 rpm for 20 min and measured protein concentration by using BCA Quantification Kit (Solarbio, Beijing, China). Cell lysates were separated by SDS-PAGE gel and transferred to polyvinylidene fluoride membranes. Blocked the membranes with 5% milk for 1 h, were incubated with antibodies against Keratin18, Bcl-2, Bax, cyclin D1, cyclin D3, cytochrome c, COX IV (CST 4850), tubulin (ac Lys40), α-Tubulin, β-Tubulin, and β-Actin at 4°C overnight then washed three times with 1 × phosphate buffered saline with 0.05% Tween-20 (PBST) buffer. Membranes were incubated with the appropriate secondary antibody and the target protein bands detected using enhanced chemiluminescence (ECL) reagent (GE Healthcare Life Science, Little Chalfont, United Kingdom) and the Amersham Image 600 (GE, Milwaukee, WI, United States).

### *Ex vivo* Pull-Down Assay

KYSE30 cell lysate were incubated with xanthohumol-Sepharose 4B beads (or only Sepharose 4B beads) in lysis buffer containing 50 mM Tris-HCl pH 7.5, 5 mM EDTA, 150 mM NaCl, 1 mM dithiothreitol, 0.01% NP-40 and 2 mg/ml bovine serum albumin at 4°C rotator, overnight. After incubation with gentle rocking, the beads were washed three times with washing buffer containing 50 mM Tris-HCl pH 7.5, 5 mM EDTA, 150 mM NaCl, 1 mM dithiothreitol and 0.01% NP-40. The binding was visualized by Western blot with KRT18 antibody.

### Knockdown and Overexpression of KRT18

For lentiviral package, *pMD2.0G*, *psPAX2*, and *shKRT18* plasmids were transfected into 293T cells using the SimpleFect transfection reagent (Signaling Dawn Biotech, Wuhan, China). The medium was changed after 12 h then cells were cultured at 37°C in a 5% CO_2_ incubator for 48 h. Viral particles were collected by filtration using a 0.45 μm filter, infected to KYSE30 cells together with polybrene (Millipore, Billerica, MA, United States) and incubated for 24 h. The medium was replaced freshly and selected with puromycin (2 mM). The effects of KRT18 knockdown on KYSE30 cells were examined by western blot and anchorage-independent cell growth assay. For the overexpression of KRT18, *pCDNA3.1-3* × *Flag-KRT18* (Youbio Biological Technology, Co., Ltd., Xi’an, China) plasmid or *pCDNA3.1-3* × *Flag* (Youbio Biological Technology, Co., Ltd., Xi’an, China) plasmid were transfected into HEK293T cell line by using SimpleFect transfection reagent for 24 h. The expression of KRT18 on HEK293T cells were examined by western blot and its effect was evaluated with anchorage-independent cell growth assay.

### Patient Derived Xenograft (PDX) Mouse Model

Female mice with severe combined immunodeficient (SCID, 6–8 weeks old) were used for these *in vivo* experiments. This study was approved by the Ethics Committee of Zhengzhou University (Zhengzhou, China). The cancer tissues were cut into small pieces and inoculated into SCID mice. After growing the tumor (∼100 mm^3^), the mice were divided into four groups as follows: (1) vehicle (*n* = 8); (2) 40 mg/kg of xanthohumol (*n* = 8); (3) 80 mg/kg of xanthohumol (*n* = 8). (4) 160 mg/kg of xanthohumol (*n* = 8). Xanthohumol was orally administered once a day for 64 days. Tumor volume was calculated from measurements of three diameters (length, width, and height) of the individual tumor base using the following formula: tumor volume (mm^3^) = (length × width × height × 0.52). Mice were monitored until tumors reached ∼1,000 mm^3^ average volume, at which time mice were euthanized and tumors extracted.

### Immunohistochemical (IHC) Analysis

Paraffin embedded tumor tissues were prepared for IHC analysis. After antigen retrieval, tissues were treated with H_2_O_2_ for 5 min and blocked with 5% goat serum, then incubated with antibodies to detect Ki-67 and KRT18 at 4°C, overnight. After incubated with second antibody, used 3,30-Diaminobenzidine to visualize the target proteins and counterstained with hematoxylin. Then the sections were photographed and analyzed using the Image-Pro Plus software (v.6.0) program (Media Cybernetics, Rockville, MD, United States).

### Statistical Analysis

All quantitative results are expressed as mean values ± SD. Used a two-tailed independent sample *t*-test to calculate the significant difference. A value of *p* < 0.05 was considered to be statistically significant.

## Results

### Xanthohumol Inhibits Proliferation of ESCC Cancer Cells

**FIGURE 1:**
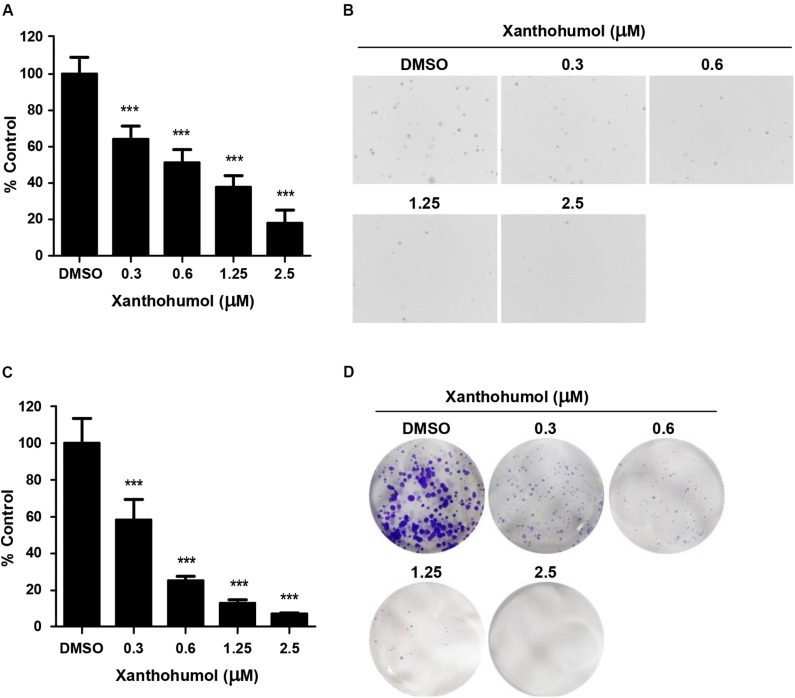
Xanthohumol inhibits the growth of ESCC cells. **(A)** Xanthohumol decreased colony formation in anchorage-independent growth of ESCC, the asterisks (*p < 0.05, **p < 0.01, ***p < 0.001) indicated a significant decrease in proliferation or colony number compared to control. **(B)** Representative photographs of the colony in soft agar. **(C)** Effects of xanthohumol on colony formation. **(D)** Representative photographs of the colony in Foci assay. Data were shown as means ± SD of values from triplicate samples and similar results were obtained from three independent experiments.

To evaluate the effect of xanthohumol on ESCC cells, we treated KYSE30 cells with xanthohumol (Figure 1). KYSE30 was derived from well-differentiated invasive ESCC resected from middle intra-thoracic esophagus of a 64-year-old Japanese man prior to treatment^1^ [Deutsche Sammlung von Mikroorganismen und Zellkulturen GmbH (DSMZ)]. Xanthohumol attenuated the anchorage-independent growth of KYSE30 cells in soft agar colony formation assay (Figure 1A). Treatment with xanthohumol significantly reduced the colony number and size as compared to control (Figure 1B). Foci assay also revealed that the number and size of colonies were significantly reduced in xanthohumol-treated group as compared to DMSO control group (Figure 1C). Figure 1D shows representative images of the colony formation in different treatment groups (Figure 1D). Cell proliferation was also inhibited after treat with xanthohumol in KYSE30 cells (Supplementary Figure S1). These results show that xanthohumol inhibits proliferation of ESCC cancer cells.

### Xanthohumol Directly Binds With KRT18, Which Is a Potential Drug Target in ESCC

**FIGURE 2:**
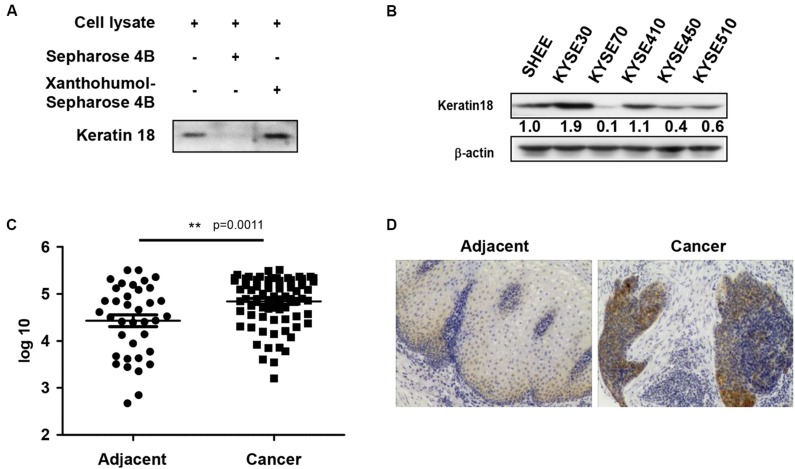
Xanthohumol binds with KRT18 and KRT18 is upregulated in ESCC. **(A)** *Ex vivo* pull-down assay showed xanthohumol bound to KRT18. **(B)** KRT18 is highly expressed in ESCC cells than that in normal cells. **(C)** The expression of KRT18 was examined by IHC analysis on an ESCC tumor tissue array. **(D)** Representative photographs of the IHC stained ESCC tumor tissues.

We identified a direct protein target of xanthohumol through the *ex vivo* pull-down assay. Incubation of KYSE30 cell lysate by adding xanthohumol-conjugated Sepharose 4B beads showed that xanthohumol was bound with KRT18 (Figure 2A). Then we evaluated the role of KRT18 in ESCC, we checked the expression of KRT18 in different ESCC cell lines and the normal SHEE esophageal cells. KRT18 was highly expressed in ESCC cell lines, especially in KYSE30 cell line (Figure 2B). To evaluate the expression of KRT18 in ESCC tissues, we checked the expression of KRT18 in an ESCC tumor microarray that included 37 pairs of adjacent tissues and cancer tissues and 40 more cancer tissues. KRT18 was significantly highly expressed in cancer tissues (Figures 2C,D).

### Xanthohumol Inhibits the Proliferation of ESCC Cells by Targeting KRT18

**FIGURE 3:**
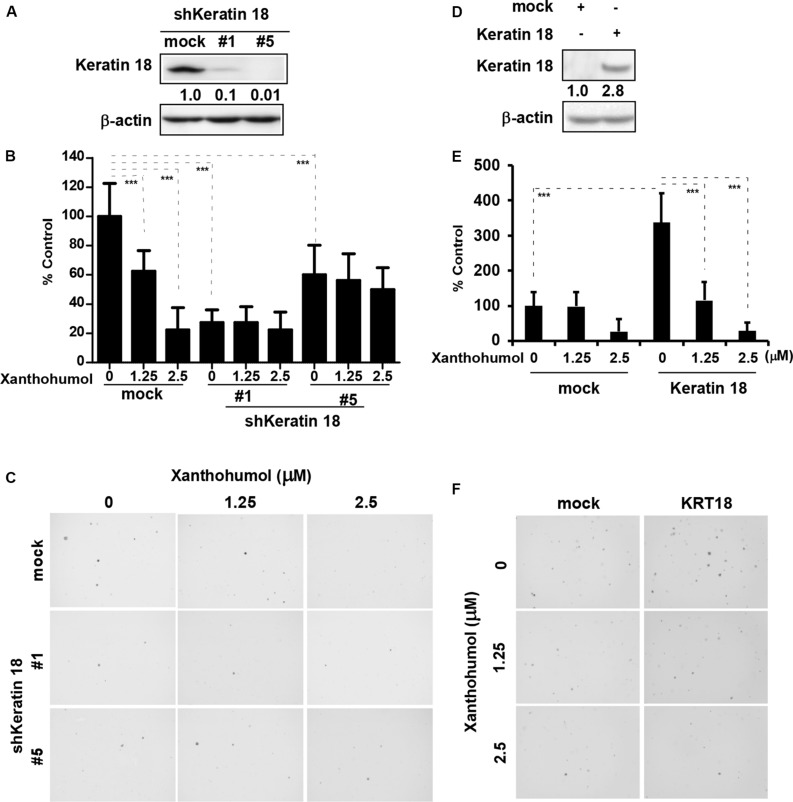
KRT18 is potential target of ESCC. **(A)** Knocking-down the expression of KRT18 in KYSE30 cell was detected by Western blotting. **(B)** Anchorage-independent cell growth was decreased after treated with xanthohumol in mock transfected cells or KRT18 knockdown cells but the colonies number did not further decrease after treated with xanthohumol in the KRT18 knockdown cells. **(C)** Representative photographs of the colonies. **(D)** KRT18 gene was overexpressed in HEK293T cells. Full length of KRT18 gene was transfect into HEK293T cells with simple-fect transfection reagent. Then it was confirmed the expression by Western blotting. **(E)** Overexpression of KRT18 promoted the colony formation in anchorage-independent cell growth, and was more sensitive to xanthohumol treatment compared to mock transfected. **(F)** Representative photographs of the colonies formed in soft agar. The asterisks (*p < 0.05, **p < 0.01, ***p < 0.001) indicated a significant difference between untreated control and xanthohumol-treated cells. Data were shown as means ± SD of values from triplicate samples and similar results were obtained from three independent experiments.

We knocked down KRT18 in KYSE30 cells by lentiviral shRNA silencing followed by selection of knockout colonies by puromycin treatment. Immunoblot analysis of KRT18 knockdown cells showed that number 1 and number 5 shRNA significantly decreased the expression of KRT18 (Figure 3A). The KRT18 knock down cells showed significantly reduced coloney formation in anchorage-independent growth assay and treatment with xanthohumol failed to further inhibit colony formation in KRT18 knock down cells (Figures 3B,C). On the other hand, overexpression of KRT18 in HEK293T cells restored the anchorage-independent colony formation, which was markedly reduced by treatment with xanthohumol (Figures 3D–F).

### Xanthohumol Induces Cell Cycle G1 Phase Arrest and Cell Apoptosis in ESCC Cells

**FIGURE 4:**
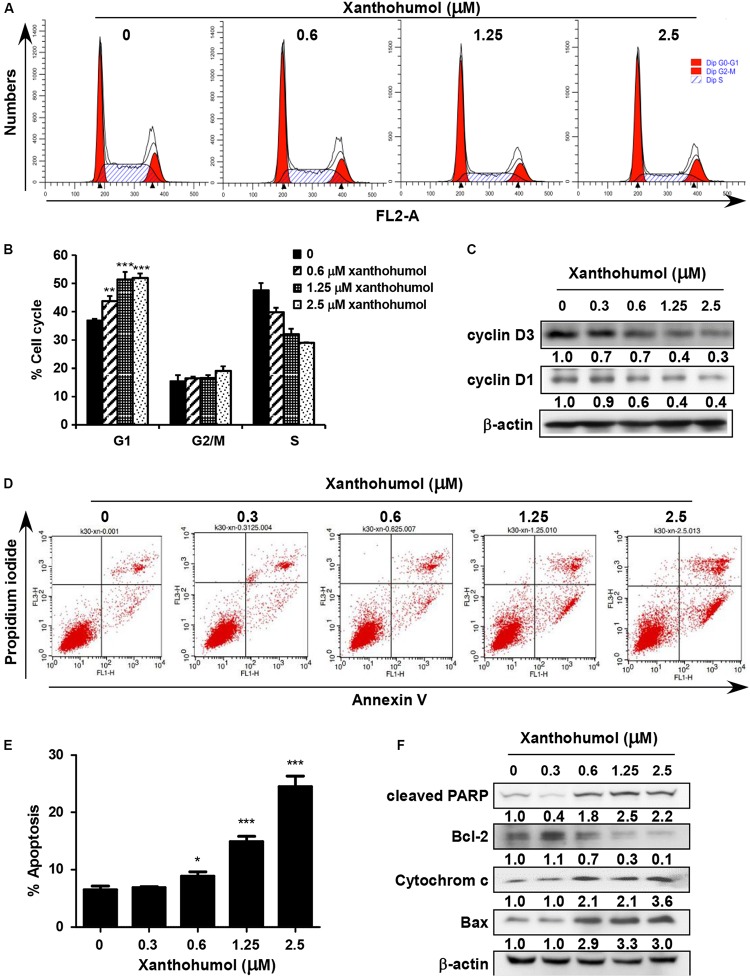
Xanthohumol induces cell cycle arrest at G1 phase and cell apoptosis. **(A)** Xanthohumol induced cell cycle arrest at G1 phase. **(B)** Xanthohumol induces cell cycle arrest at G1 phase in a concentration dependent manner. **(C)** Western blotting results showed the decreased expression of cyclinD1 and cyclinD3, G1 phase marker for cell cycle. **(D)** Xanthohumol treatment induced cell apoptosis. **(E)** Xanthohumol treatment can induce cell apoptosis in a concentration dependent manner. **(F)** Xanthohumol upregulated the expression of cell apoptosis markers like cleaved-PARP, Bcl-2, cytochrome c and Bax by determined with Western blotting. The asterisks (*p < 0.05, **p < 0.01, ***p < 0.001) indicated a significant difference between untreated control and xanthohumol -treated cells. Data were shown as means ± SD of values from triplicate samples and similar results were obtained from three independent experiments.

We examined the effect of xanthohumol on cell cycle and cell apoptosis. Treatment with xanthohumol for 24 h caused significant G1 phase cell cycle arrest and decreased cyclinD1, cyclinD3 expression in KYSE30 cells (Figures 4A–C). In addition, cells were treated with xanthohumol for 48 h to assess cell apoptosis. The annexinV + /PI + staining revealed that xanthohumol (2.5 μM) treatment induced apoptosis, which was supported by Figures 4D,E the increased expression of cleaved-PARP, cytochrome c and Bax, and inhibition of Bcl-2 in a concentration-dependent manner (Figure 4F). We also checked the location of cytochrome c after treat with xanthohumol, it was downregulated in mitochondrial protein lysate and upregulated in cytosolic proteins(Supplementary Figure S3)

### Xanthohumol Functions Through Promotion of KRT18 Degradation

**FIGURE 5:**
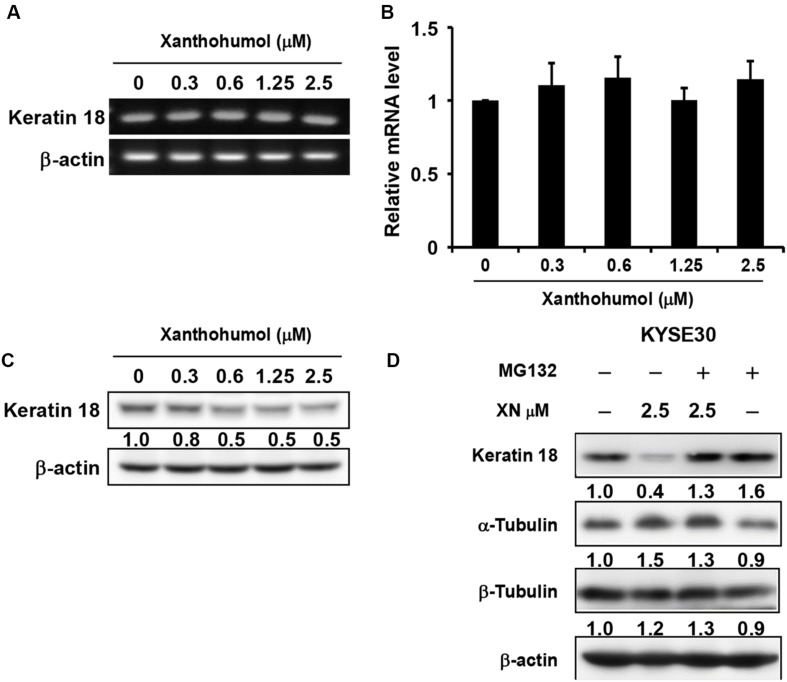
Xanthohumol promotes the degradation of KRT18 protein. **(A)** The mRNA level of KRT18 was examined after treated with xanthohumol for 48 h by RT-QPCR, AGE (agarose gel electrophoresis) results showed the intensity of KRT18 mRNA and β-Actin. **(B)** RT-QPCR results showed the mRNA level of KRT18 was not changed by xanthohumol treatment. **(C)** Western blotting results showed xanthohumol treatment decreased the protein level of KRT18. **(D)** MG132 treatment restored xanthohumol-induced KRT 18 degradation.

We then explored the mechanisms by which xanthohumol inhibits ESCC cell proliferation through targeting KRT18. We first checked the mRNA level of KRT18 after treatment of cells with xanthohumol (Figures 5A,B). Results showed that xanthohumol did not influence the mRNA level of KRT18 (Figures 5A,B). Then we examined the KRT18 protein level after incubating cells with xanthohumol. The results revealed that xanthohumol decreased KRT18 protein expression and it was restored by MG132 pre-treatment (Figures 5C,D). These results indicated that xanthohumol may exert antiproliferative effects by enhancing KRT18 degradation.

### Xanthohumol Inhibits Patient-Derived Xenograft ESCC Growth *in vivo*

**FIGURE 6:**
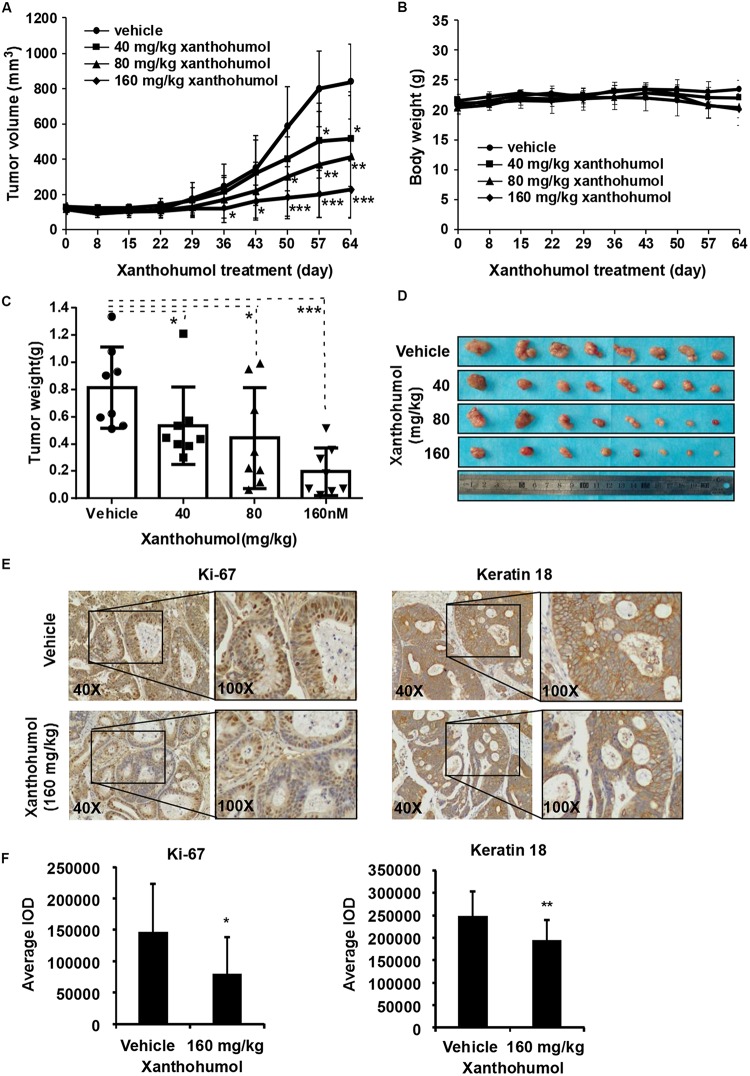
Effects of xanthohumol on patient-derived xenograft (PDX) tumor growth *in vivo*. **(A)** Xanthohumol treatment decreased the tumor volume compared to vehicle treatment. **(B)** Xanthohumol treatment had no effect on mice body weight. **(C)** Tumor weight was decreased after xanthohumol treatment compared to vehicle treatment. **(D)** Representative photographs of the tumors. **(E)** The expression of Ki-67 and KRT18 was examined by IHC analysis. Representative photographs showed the expression of these two proteins. **(F)** Quantitation of Ki-67 and KRT18 expression from the IHC results. Data are expressed as IOD values ± SD. The asterisks (*p < 0.05, **p < 0.01) indicated a significant decrease in Ki-67 and KRT18 expression in xanthohumol treated tissues compared to untreated controls.

In order to explore the anti-tumor effects of xanthohumol *in vivo*, we used PDX tumor model in mice. We chose one patient case LEG32 to conduct experiment because KRT18 was highly expressed in LEG32 tumor tissues (Supplementary Figure S2). Cancer tissues were cut into small pieces and inoculated in to SCID mice. When the tumor volume is about 100 mm^3^, tumor-bearing mice were divided into four groups, each group containing eight mice. Vehicle or xanthohumol (40, 80, or 160 mg/kg body weight) was administered by gavage once a day for 64 days. Results indicated that xanthohumol significantly decreased the tumor growth relative to the control group without causing any change in mouse body weight (Figures 6A–D). Then we examined the expression of Ki67 and KRT18 in xenograft tumor sections using the IHC analysis. Xanthohumol remarkably suppressed the expression of both Ki67 and KRT18 protein in tumor tissues isolated from xanthohumol-treated group as compared to vehicle- treated group (Figures 6E,F).

## Discussion

Esophageal cancer is a remarkable public health issue, especially in developing countries. Although a number of FDA approved therapies, such as growth factor receptor inhibitors and immunotherapies are currently used in clinical settings, none of these therapies are uniformly effective in all patient groups. Thus the hunt for novel therapies remains a constant venture to the research scientists (Shi et al., 2018; Song et al., 2018, 2019; Xu et al., 2018; Ji et al., 2019).

Because of the diverse biological activity and low systemic toxicity, many naturally occurring plant-derived chemicals have attracted considerable attention for new drug development. Xanthohumol, a prenylated flavonoid isolated from *hops*, has been reported to possess antibacterial, antiplatelet, anti-obesity and anticancer effects. It has been reported that xanthohumol inhibits colorectal cancer cells via downregulation of Hexokinases II-mediated glycolysis (Liu W. et al., 2019; Logan et al., 2019), exhibits anti-myeloma activity *in vitro* through inhibition of cell proliferation via the ERK and JNK-dependent mechanism (Slawinska-Brych et al., 2019) and exerts anticancer effects against gastric cancer (Wei et al., 2018). However, the roles of xanthohumol in ESCC were yet to be fully elucidated. Liu X. et al. (2019) reported that xanthohumol inhibits ESCC through blockade of Akt kinase activity. These authors also demonstrated that xanthohumol inhibited proliferation of ESCC cells having low Akt level thereby indicated that there might have additional mechanisms underlying anticancer effect of xanthohumol. In the present study, we have found that treatment of KYSE30 cells with xanthohumol resulted in significant inhibition of anchorage-independent colony formation (Figures 1A–D) and induction of G1 phase cell cycle arrest and apoptosis (Figures 4A–D). The alterations in the expression of apoptosis markers such as increased PARP cleavage, cytochrome c release and Bax induction along with inhibition of Bcl-2 expression further support the antitumor effect of xanthohuol on ESCC cells. We further identified KRT18 as a new target of xanthohumol, which directly binds with KRT18 (Figure 2A) and reduced the protein expression of KRT18 without affecting its mRNA transcript (Figures 5A–D). Moreover, shRNA-mediated knock down of KRT18 in KYSE30 cells reduced the colony formation and treatment of these cells with xanthohumol failed to further suppress the growth of KYSE30 cells. Conversely, overexpression of KRT18 enhances anchorage-independent growth, which was diminished by treatment with xanthohumol (Figures 3A–F). These data indicate xanthohumol is a potential chemotherapeutic compound for ESCC, and KRT18 is a primary target of xanthohumol.

Keratin 18 (KRT18) is a member of the intermediate filament family of cytoskeletal protein. Its expression is upregulated in ESCC tissues (Kilic-Baygutalp et al., 2016). However, the implication of elevated expression of KRT18 in ESCC cells is not clear. It has been reported that high Keratin18 predicts aggressive hepatocellular cancer phenotype (Golob-Schwarzl et al., 2019) and serves as a diagnostic and prognostic factor for acute alcoholic hepatitis (Vatsalya et al., 2019). In contrast, Bettermann et al. (2016) have demonstrated that KRT18 leads to the development of steatohepatitis and liver carcinogenesis in old-age mice. Thus, the role of KRT18 in hepatocellular carcinoma requires further study. Several other studies have shown that KRT18 plays an important role in gastric cancer, colorectal cancer, prostate cancer, and breast cancer (Goliwas et al., 2017; Jedroszka et al., 2017; Peduk et al., 2018; Zhang et al., 2019). In our present study, we found KRT18 is highly expressed in ESCC cell lines and ESCC tissues compared with normal tissues (Figures 2B–D). However, further studies are warranted to elucidate the mechanism underlying elevation of KRT18 during tumorigenesis.

Patient-derived tumor xenografts (PDXs) engrafted into immune-compromised rodents such as SCID mice is a useful preclinical model of human tumor growth. PDX models are used to screen for biomarkers and predict clinical trial drug response, because these models conserve original tumor characteristics such as heterogeneity, complexity, and molecular diversity (Kopetz et al., 2012; Tentler et al., 2012; Nunes et al., 2015; Park et al., 2016). Therefore, we also evaluated the effect of xanthohumol on the growth of ESCC PDX tumors in mice. Compared with control group, the tumor volume and tumor weight in the xanthohumol-treated mice were significantly decreased (Figures 6A–D). We also checked the expression of Ki67 and KRT18 in the tumors by using IHC assay, which revealed that both the markers are downregulated in the xanthohumol-treated tumors (Figures 6E,F). The PDX models can supply important information for the translation from preclinical studies to clinical studies. In summary, our study suggested that xanthohumol can inhibit the progression of ESCC, at least in part by binding with KRT18 and promoting KRT18 degradation.

## Data Availability Statement

The datasets generated for this study are available on request to the corresponding author.

## Ethics Statement

The animal study was reviewed and approved by Ethics Committee of Zhengzhou University (Zhengzhou, China).

## Author Contributions

SY, MS, M-HL, and ZD are involved in study concept and design, acquisition of data, analysis and interpretation of data, and drafting of the manuscript. SY, MS, RZ, XL, WK, JL, and YK performed experiments analysis and interpretation of data. KL, CZ, and J-HS have supported the materials. SY, M-HL, and ZD wrote the manuscript. ZD and M-HL had supervision of all study.

## Conflict of Interest

The authors declare that the research was conducted in the absence of any commercial or financial relationships that could be construed as a potential conflict of interest.

## Footnotes

1 https://www.dsmz.de/
