# Supplementary figures and images for: Xanthohumol Inhibits the Growth of Keratin 18-Overexpressed Esophageal Squamous Cell Carcinoma in vitro and in vivo
Source: Front Cell Dev Biol. 2020 May 19;8:366. doi: 10.3389/fcell.2020.00366 (PMC7248302; doi:10.3389/fcell.2020.00366)

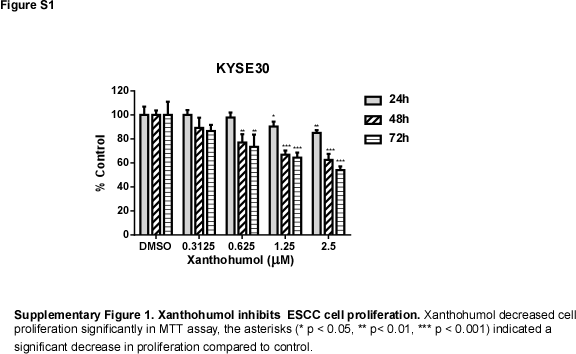

Supplement: Supplementary file 1 [file Image_1.TIF]

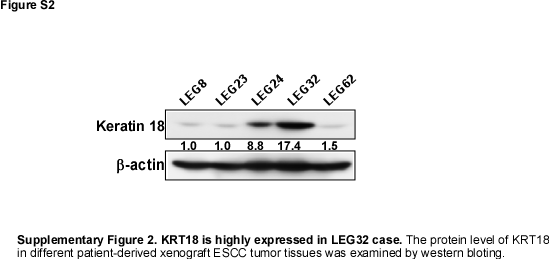

Supplement: Supplementary file 2 [file Image_2.TIF]

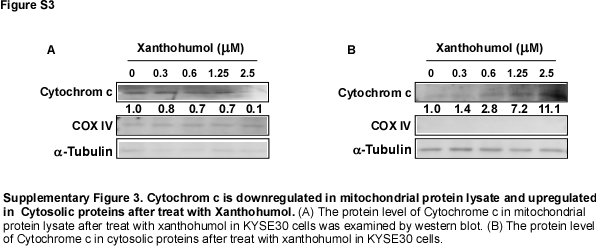

Supplement: Supplementary file 3 [file Image_3.TIF]
